# ITC-Net-MingledApp: A comprehensive dataset of mixed mobile application traffic for network traffic classification in diverse environments

**DOI:** 10.1016/j.dib.2026.112731

**Published:** 2026-03-30

**Authors:** Abolghasem Rezaei Khesal, Mohammad Sadeq Abed, Mohammad Mahdi Mohammadi, Mohammad Mahdi Mahdavirad, Ramtin Kabiri, Mehdi Teimouri

**Affiliations:** Information Theory and Coding (ITC) Laboratory, University of Tehran, Tehran, Iran

**Keywords:** Network traffic analysis, Traffic classification, Application identification, Mobile-app fingerprinting, Encrypted traffic, Robustness, Domain adaptation, Raw labelled dataset

## Abstract

This dataset was collected from 36 popular Android applications across three distinct network scenarios. The scenarios varied by Internet service provider (ISP), geographic location, and individual users. Traffic was generated through human interactions on physical devices for 5–15 minutes using multiple smartphones simultaneously to simulate real traffic from a network supervisor's perspective. The traffic capture method did not require root privileges and filtered out any background traffic. The dataset includes approximately 35 million packets, 399 K bidirectional flows, and 29 GB of data. This dataset allows for evaluating model performance under diverse conditions and testing domain adaptation scenarios. The dataset is provided in raw format (PCAP files), to offer researchers flexibility in developing models based on different traffic objects, features, and methods.

Specifications Table

**Table:** 

Subject	Computer Sciences
Specific subject area	Classifying mobile application traffic is challenging due to its diversity and dynamism, including network conditions, user behaviour, and application updates. Accurate identification and classification of concurrent traffic from multiple applications is essential for generalizing network traffic classifiers and ensuring their robustness in dynamic environments like mobile networks. However, current datasets are often limited to single network environments and capture application traffic one at a time, limiting their usefulness in assessing a model's robustness and compatibility.
Type of data	Raw, Analysed, Filtered, Processed
Data collection	We used a laptop and at least three smartphones running the applications to capture traffic. The laptop, which had Windows 10 and an internal dual-band network card, was connected to the internet and shared its connection with the smartphones via a hotspot. *Wireshark* was configured to capture traffic through the laptop’s shared ``Local Area Connection'' interface, allowing smartphones to access the internet while their network traffic was recorded. However, this capture included significant background traffic, and different applications' traffic was not separable. To isolate each application’s network traffic, we installed *PCAPdroid* on the smartphones in non-root mode and ran Wireshark and PCAPdroid simultaneously. After collecting the traffic data, we separated the application traffic from the background traffic by comparing IP addresses and ports identified by both tools. For each Wireshark trace, we isolated the traffic for each application based on its server's IP address and port, removing any pairs in Wireshark that did not match the corresponding pairs in the PCAPdroid. This method was implemented in *Python 3* using the *Scapy* library, and the code can be found in the supplementary materials.
Data source location	The traffic generation process was conducted by three teams from ITC-LAB over five months, from March 25 to August 25, 2024, operating across three geographically separated locations: Iraq-Baghdad Province, Iran-Tehran and Iran-Qom provinces. The resulting dataset has been archived in the Mendeley Data repository system. Due to storage constraints inherent to the platform’s architecture, the complete dataset is distributed across **five interconnected repository entries**, each containing logically partitioned segments of the collected dataset.
Data accessibility	Repository name: ITC-Net-MingledApp Data identification number: 10.17632/96jwzrp7fd.1, 10.17632/9frgkybxhn.1, 10.17632/4b9xpz4gd3.1, 10.17632/zsffy3j9y6.1, 10.17632/23v73hh7r5.1 Direct URL to data: https://data.mendeley.com/datasets/96jwzrp7fd/1, https://data.mendeley.com/drafts/9frgkybxhn/1, https://data.mendeley.com/datasets/4b9xpz4gd3/1, https://data.mendeley.com/datasets/zsffy3j9y6/1, https://data.mendeley.com/datasets/23v73hh7r5/1
Related research article	Khesal, Abolghasem Rezaei and Teimouri, Mehdi, Interpretable Feature Engineering for Robust Mobile Application Traffic Classification. Available at SSRN: https://ssrn.com/abstract=6293191 or 10.2139/ssrn.6293191 [1].

## 1 Value of the Data

• This dataset allows for evaluating model performance under diverse conditions and testing model generalization scenarios like intra-dataset and inter-dataset generalization tests.
• Generated through real human interactions on actual smartphones using a non- rooted method, the dataset is more representative and suitable for mobile app traffic analysis compared to synthetic data.
• Existing traffic classification datasets have mostly been collected in a single, invariant network environment. Only some limited datasets include data from varying network scenarios—such as different times, devices, or app versions. however, these datasets have their own limitations and are often only partially accessible. On the other hand, their collection methodology typically involves capturing one app at a time, whereas from a network observer or manager's perspective real network traffic consists of mixed packets from multiple devices and applications simultaneously. Our new dataset, ITC-MingledApp, was specifically collected to provide realistic traffic data that a network supervisor would encounter at any link or hub. This dataset reflects mixed packets from various sources sent concurrently on the same channel. By making it publicly available, we aim to support the development of robust application identification solutions, domain adaptation, and model generalization.
• The dataset is published in its original raw PCAP (Packet Capture) file format, ensuring that researchers retain full access to the unprocessed network traffic data. This format preserves the complete packet-level information, including headers and payloads, thereby enabling detailed forensic and analytical investigations.

## 2 Background

The growing use of mobile apps has heightened the demand for reliable app identification solutions, essential for effective network management and security. Additionally, these solutions provide valuable profiling information for advertisers, insurers, and security agencies [[12], [13], [14], [15]]. This increased importance has led to significant interest and extensive research in academia and industry.

Robustness and domain adaptation remain significant challenges in traffic classification and application identification. Recent studies [2,3,16] have shown that while current classifiers perform well with conventional machine learning methods, they often suffer substantial performance declines when tested on different datasets, indicating a lack of robustness in practical networks. This issue stems from the unpredictable nature of real-world network environments [16] and the dynamic behaviour of mobile apps [2,3,13]. To overcome these challenges, it is essential to have a diverse dataset of realistic traffic samples from various network scenarios. However, most existing datasets have been collected in a single, static network environment [2,7,10] and focus on one application at a time [11,17], failing to accurately represent multi-application network traffic.

Existing traffic classification datasets, as listed in Table 1, have mostly been collected in a single, invariant network environment. Only Appscanner [3], Cross Market [8], CrossNet2021 [2], and ITC-net-60 [11] include data from varying network scenarios—such as different times, devices, or app versions. however, these datasets have their own limitations and are often only partially accessible. On the other hand, their collection methodology typically involves capturing one app at a time, whereas from a network observer or manager's perspective real network traffic consists of mixed packets from multiple devices and applications simultaneously.

**Table 1:** Summary of available datasets.

Dataset	Mobile Apps Traffic	No. Apps	No. Real Human Users	No. Device	Capture Span	Capture Session Duration	Released Data	No. Network Environments / Scenarios	Shared data volume (Bytes)
CrossNet2021 [2]		20	N/A	N/A	N/A	N/A	PCAP files	2	2.1 GB
Appscanner [3]	•	110	- (simulation)	2	N/A	30 min	Feature set	8	N/A
Unicauca [4]		78	N/A	N/A	Six days in 2017	N/A	Feature set	1	N/A
Mobilegt [5]	•	12	10	N/A	October 2016 - March 2017	16 min	Feature set	1	N/A
Andrubis [6]	•	1M	- (simulation)	N/A	2012.06.13 - 2016.03.25	N/A	PCAP files	1	N/A
Mirage [7]	•	40	280	1	May 2017 - May 2019	5 - 10 min	Feature set	1	N/A
Cross Market [8]	•	400	N/A	N/A	2017.08.28 - 2017.11.20	N/A	PCAP files	3	≈ 2 MB
UTMobileNet2021[9]	•	16	- (simulation)	3	N/A	N/A	PCAP files	N/A	N/A
ISCX2016 [10]	•	N/A	N/A	N/A	N/A	N/A		N/A	N/A
ITC-Net-Blend-60 [11]	•	60	5	7	October - December 2021	3 - 15 min	PCAP files	5	35.91 GB
**ITC-MingledApp**	•	**36**	**5–8**	**>10**	**March-August 2024**	**3 - 15 min**	PCAP files	**3**	**28.73 GB**

* Cells marked as “N/A” indicate that the corresponding information is not reported in the original dataset publications.

The Appscanner dataset is available only as a statistical feature set, limiting its practical application. In contrast, the Cross Market and CrossNet2021 datasets are provided in raw form but are constrained by their small size and the limited number of apps included, making them unsuitable for methods like deep learning. This small sample size can also compromise validation accuracy, as the number of applications directly affects model performance. Notably, the CrossNet2021 dataset contains just 20 Chinese apps and lacks representation of international apps, such as YouTube.

Our dataset, ITC-MingledApp, was specifically collected to provide realistic traffic data that a network supervisor would encounter at any link or hub. This dataset reflects mixed packets from various sources sent concurrently on the same channel. By making it publicly available, we aim to support the development of robust application identification solutions, domain adaptation, and model generalization.

## 3 Data Description

The dataset is systematically organized into **three distinct network scenarios**, each corresponding to a specific geolocation. Each network scenario is housed within a dedicated, separate repository. Table 2 presents number of packets, bytes, flows, and capture duration for each network scenario. This modular design ensures clear segregation of data based on its geographical and operational context, allowing researchers to isolate or compare environmental variables effectively.

**Table 2:** Dataset specifications per network scenario.

Scenario	No. PCAP file	No. Packets	No. Bytes (GB)	No. Bi-Flows*	Capture Duration (Minute)	Android Version
Iran-Qom	159	11,353,374	8.61	183,139	2135	12, 14
Iran-Tehran	225	16,655,362	13.80	166,039	2254	10, 13, 14
Iraq-Baghdad	153	6864,772	6.32	49,807	1719	10 - 14
Total	**537**	**34,873,508**	**28.73**	**398,985**	**6109 (≈102 hour)**	

* The “1-second threshold” is interpreted as the inactivity timeout used for flow termination, meaning that a flow is split when no packets are observed for a duration of one second.

Within each scenario repository, the data is packaged into dedicated compressed archives. Each archive contains PCAP files that adhere to a strict, informative naming convention:<Trace_Timestamp>_<Application_Name>_<Download_Upload_Speed>.pcap

This convention embeds three critical metadata dimensions directly into the filename:

• Temporal Context (Trace_Timestamp): The precise time of the capture.
• Application Context (Application_Name): The specific application generating the traffic.
• Network Condition (Download_Upload_Speed): The recorded network bandwidth profile during the capture session, enabling research on performance under constrained or variable connectivity. The download and upload speeds were measured using the Speedtest application on the laptop and smartphones prior to each capture session and were used to label the corresponding traffic traces.

A detailed description of the captured traffic in each scenario, along with supplementary information, is provided in Table A1 for each application and in Table A2 for each capture, found in the Appendix. The complete corpus comprises **537 individual PCAP traces**, amounting to **28.7 GB** of raw network traffic data.

It should be noted that part of the dataset was collected in regions with specific network policies, such as filtering and traffic throttling. As a result, certain traffic characteristics may differ from those observed in less restricted network environments. While this may limit direct generalization to all global network conditions, it also reflects realistic usage scenarios in constrained environments and provides valuable data for studying network behavior under such conditions.

## 4 Experimental Design, Materials and Methods

The dataset collection methodology included three main stages: Application Selection, Traffic Capture Setup, and Traffic Generation.

### 4.1 Application selection

To collect traffic data, the first step was to select applications for monitoring from the vast array available. We chose 36 Android applications from the top free apps in the Google Play Store and two major Iranian app markets, Cafe Bazaar [18] and Myket [19]. Our selection criteria included:

• The apps must require internet connectivity for their core functions,
• Generate traffic through user interactions,
• Be part of other datasets like ITC-Net-Blend60 or ISCX2016 or be newly popular apps.

These 36 apps span 15 distinct categories, as listed in Table 3.

**Table 3:** List of the applications in the dataset.

#	Category	Name	Package Name
1	**AI_Chat**	Copilot	com.microsoft.copilot
2		Perplexity	ai.perplexity.app.android
3	**Browser**	Google Chrome	com.android.chrome
4		Microsoft Edge	com.microsoft.emmx
5		Opera Mini	com.opera.mini.native
6	**Communication**	Skype	com.skype.raider
7		Telegram	org.telegram.messenger
8		Whatsapp Messenger	com.whatsapp
9		Eitaa	ir.eitaa.messenger
10		Gmail	com.google.android.gm
11		Email	com.samsung.android.email.provider
12	**Education**	Duolingo	com.duolingo
13		Elsa Speak	us.nobarriers.elsa
14	**Entertainment**	Youtube	com.google.android.youtube
15		Filimo	com.aparat.filimo
16	**Multifunctional**	Rubika	app.rbmain.a
17		Snapp	cab.snapp.passenger
18	**Crypto Marketing**	CoinMarketCap	com.coinmarketcap.android
19	**Game**	Clash of Clans	com.supercell.clashofclans
20		Mencherz	com.incyteltech.mencherz
21	**Maps & navigation**	Neshan	org.rajman.neshan.traffic.tehran.navigator
22		Google Maps	com.google.android.apps.maps
23	**Music & Audio**	Radio Javan	com.radiojavan.androidradio
24		Spotify	com.spotify.music
25	**Photography**	Canva	com.canva.editor
26	**Cloud Storage**	Google Drive	com.google.android.apps.docs
27		OneDrive	com.microsoft.skydrive
28	**Shopping**	Alibaba.com	com.alibaba.intl.android.apps.poseidon
29		Digikala	com.digikala.diagon
30		Torob	ir.torob
31	**Social**	LinkedIn	com.linkedin.android
32		Instagram	com.instagram.android
33		Facebook lite	com.facebook.lite
34		Facebook	com.facebook.katana
35	**Android app market**	Google Play Store	com.google.vending
36		Bazaar	com.farstitel.bazaar

### 4.2 Traffic capture setup

**Fig. 1:**
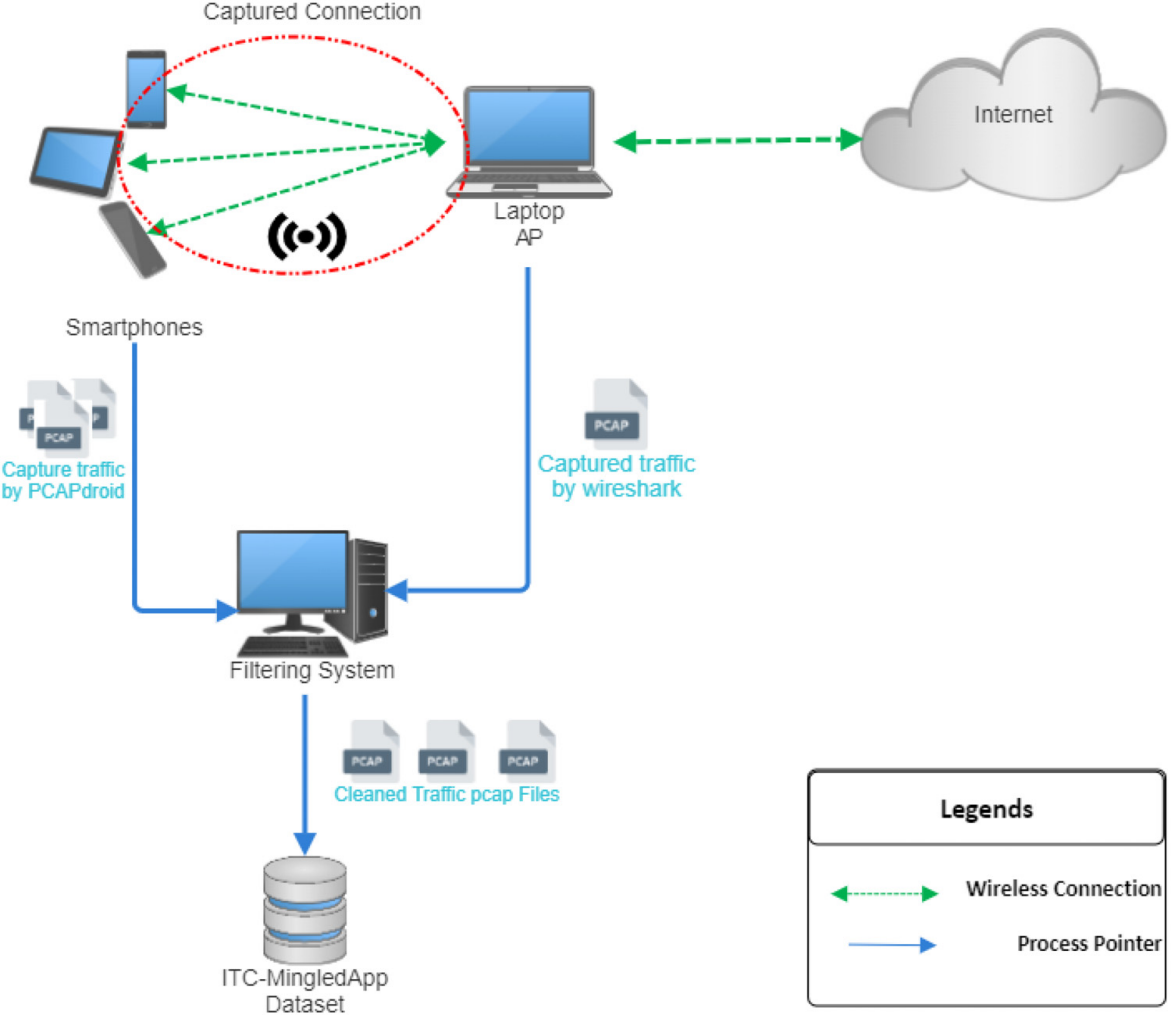
Traffic capture setup.

We used a laptop and at least three smartphones running the applications to capture traffic (Fig. 1). The laptop, which had Windows 10 and an internal dual-band network card, was connected to the internet and shared its connection with the smartphones via a hotspot. *Wireshark* [20] was configured to capture traffic through the laptop’s shared ``Local Area Connection'' interface, allowing smartphones to access the internet while their network traffic was recorded. However, this capture included significant background traffic, and different applications' traffic was not separable. To isolate each application’s network traffic and obtain application-level flow labeling, we installed PCAPdroid [21] on the smartphones in non-root mode and ran Wireshark and PCAPdroid simultaneously. After collecting the traffic data, we separated the application traffic from the background traffic by comparing IP addresses and ports identified by both tools. For each Wireshark trace, we isolated the traffic for each application based on its smartphone's IP address, retaining only those flows in Wireshark that matched the corresponding IP-port pairs identified by PCAPdroid, while removing unmatched flows as background traffic.. This method was implemented in *Python 3* using the *Scapy* library, and the code can be found in the supplementary repository [22].

To validate the accuracy of the filtering process, we performed a consistency analysis between the flows identified by PCAPdroid and those extracted from Wireshark. Only flows with matching IP-port pairs in both tools were retained. Furthermore, a subset of the captured traces was manually inspected to verify that the retained flows correspond to the intended application behavior and that unrelated background traffic was effectively removed. This process confirmed a high level of agreement between the two sources.

**Fig. 2:**
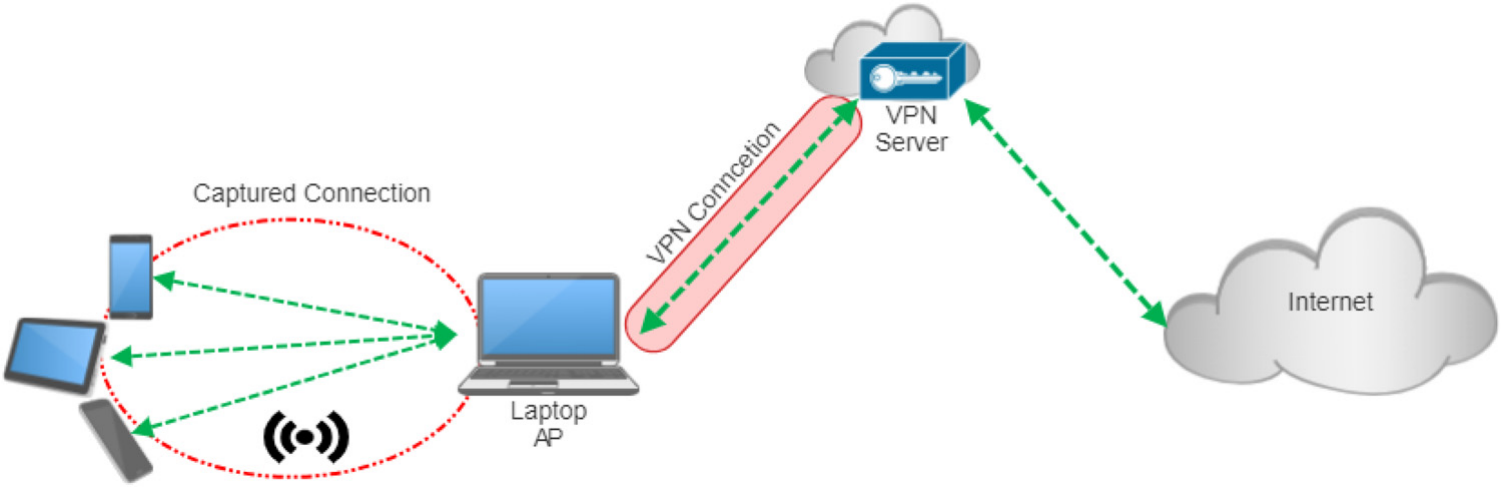
Capture setup with VPN.

Some applications used in our study imposed access restrictions in Iran, necessitating the use of a VPN connection for proper execution. To address this, we configured a VPN on the laptop while performing traffic capture at the network interface level prior to VPN tunneling. (Fig. 2). In this way, the recorded packets enabled the capture of application traffic, including those requiring VPN connectivity. While traffic was captured before VPN encapsulation, we note that VPN client configurations on the host machine may still influence certain packet-level characteristics, such as MTU and timing. Therefore, minor variations in these features may be present in the recorded traffic. This aspect reflects realistic network conditions in restricted environments where VPN usage is common and is considered a potential limitation when generalizing the dataset to other network settings.

### 4.3 Traffic generation

The traffic generation process was conducted by three teams from ITC-LAB over five months, from March 25 to August 25, 2024. Each team collected traffic from a different network scenario. Before data collection, team members were informed about the objectives of traffic capture and public data release in PCAP format, and they received training on traffic collection. Each team was responsible for simultaneously capturing traffic from at least three applications, with volunteers interacting with a single app on a specific smartphone for 5 to 15 min while using the apps normally to explore their functionalities.

Due to storage limitations in the Mendeley Data repository, the dataset is stored in five linked repositories based on the region and scenario of data collection [[22], [23], [24], [25], [26]]. The mapping between repositories and network scenarios is as follows:

- Scenario 1 (Tehran): Tehran Dataset #1 [24] and Tehran Dataset #2 [25],

- Scenario 2 (Qom): Qom Dataset [23],

- Scenario 3 (Iraq): Iraq Dataset [26],

- Supplementary materials: Additional data and code [22].

## Limitations

• This dataset only includes traffic of a specific subset of Android applications. It does not include traffic from other operating systems or applications.
• Each capture session is represented by a compressed RAR file that contains PCAP file per application.
• Each capture session was conducted for a fixed duration of between 5 and 15 min, although the duration of each PCAP file may vary due to the removal of background traffic.
• Due to internet access restrictions in Iran, some captures were recorded using a VPN; however, the packets were captured before entering the VPN connection. (For more details, please refer to the Supplementary Materials.)
• There is a possibility that learned models may capture subtle VPN-related artifacts (“VPN fingerprints”) in addition to application-specific traffic patterns. Researchers should consider this factor when interpreting classification performance and generalizing results to non-VPN environments.
• Due to storage limitation in Mendeley Data repository, the dataset stored in 5 linked repositories based on the regional data collection.

## Ethics Statement

The network traffic in the ITC-Net-MingledApp dataset was generated in a controlled experimental environment using temporary test accounts created specifically for the study on several mobile applications, including Telegram. The activities were performed by volunteer participants using experiment devices, and communications occurred only between these test accounts within the experimental setup. No real personal accounts or external users were involved in the data generation process. The released dataset contains only anonymized network-level traffic traces, and no personally identifiable information (PII) or sensitive user content is included. All test accounts used during the experiments were deleted after the completion of data collection. According to the applicable research policies at University of Tehran, the study did not require formal ethics committee approval because no identifiable personal data were collected or retained.

## CRediT Author Statement

**Mehdi Teimouri:** Conceptualization, Methodology, Supervision, Writing – review & editing; **Abolghasem Rezaei Khesal:** Methodology, Software, Data curation, Investigation, Validation, Writing – original draft, Writing – review & editing; **Mohammad Sadeq Abed:** Investigation, Data curation; **Mohammad Mahdi Mohammadi:** Investigation, Data curation; **Mohammad Mahdi Mahdavirad:** Investigation, Data curation; **Ramtin Kabiri:** Investigation, Data curation.

## Declaration of generative AI and AI-assisted technologies in the manuscript preparation process

In the drafting phase of this manuscript, generative language models, specifically Perplexity.ai and ChatGPT, were employed as tools to refine the linguistic expression and enhance readability for a native English-speaking audience. It is important to emphasize that these tools were utilized solely for stylistic refinement. The authors maintained rigorous oversight throughout the process, meticulously reviewing and editing all generated content. Consequently, the authors affirm their complete responsibility for the intellectual content and accuracy of the information presented in this publication.

## Figures and Tables

**Table A1:** Dataset specifications per application.

#	Name	Scenario	No. Packets	No. Bytes (MB)	No. Bi-Flows	Capture Duration (Minute)
1	Copilot	Iran-Qom	203,098	140	3130	50
		Iran-Tehran	160,170	114	5265	49
		Iraq-Baghdad	104,695	73.3	1616	30
2	Perplexity	Iran-Qom	126,663	63.4	4007	50
		Iran-Tehran	148,256	91.2	3575	60
		Iraq-Baghdad	133,401	92	2635	74
3	Google Chrome	Iran-Qom	267,182	195	9203	51
		Iran-Tehran	392,082	260	20,335	73
		Iraq-Baghdad	134,411	86.2	3954	76
4	Microsoft Edge	Iran-Qom	332,090	254	6790	39
		Iran-Tehran	488,199	400	8341	60
		Iraq-Baghdad	178,280	138	2523	24
5	Opera Mini	Iran-Qom	239,464	168	13,045	37
		Iran-Tehran	183,067	136	6392	40
		Iraq-Baghdad				
6	Skype	Iran-Qom	265,469	190	7264	74
		Iran-Tehran	131,711	76.2	4062	39
		Iraq-Baghdad	251,747	145	2339	42
7	Telegram	Iran-Qom	222,543	146	10,824	59
		Iran-Tehran	271,311	197	3120	60
		Iraq-Baghdad	141,750	116	1464	115
8	Whatsapp Messenger	Iran-Qom	178,815	147	1635	49
		Iran-Tehran	337,795	234	1883	99
		Iraq-Baghdad	39,132	10.1	323	24
9	Eitaa	Iran-Qom	94,813	61.4	4193	36
		Iran-Tehran	1246,318	1030	4287	102
		Iraq-Baghdad				
10	Gmail	Iran-Qom	296,817	245	3360	86
		Iran-Tehran	204,163	158	3516	69
		Iraq-Baghdad	944,708	989	2578	116
11	Email	Iran-Qom	141,900	94.2	4377	89
		Iran-Tehran	8188	5.32	357	21
		Iraq-Baghdad				
12	Duolingo	Iran-Qom	90,451	62.2	3527	83
		Iran-Tehran	146,163	98.8	5119	92
		Iraq-Baghdad	118,614	98.6	1573	82
13	Elsa Speak	Iran-Qom	68,456	41.8	2862	53
		Iran-Tehran	283,765	203	4996	73
		Iraq-Baghdad				
14	Youtube	Iran-Qom	291,874	220	13,900	69
		Iran-Tehran	522,720	595	3056	49
		Iraq-Baghdad	578,422	603	3488	94
15	Filimo	Iran-Qom	1599,569	1480	3055	96
		Iran-Tehran	1649,947	1300	3552	98
		Iraq-Baghdad				
16	Rubika	Iran-Qom	850,034	712	7205	63
		Iran-Tehran	903,427	663	9109	30
		Iraq-Baghdad				
17	Snapp	Iran-Qom	156,423	104	4410	51
		Iran-Tehran	217,561	161	5593	58
		Iraq-Baghdad				
18	CoinMarketCap	Iran-Qom	171,684	111	7462	66
		Iran-Tehran	342,052	241	7848	50
		Iraq-Baghdad				
19	Clash of Clans	Iran-Qom	822,640	525	6460	57
		Iran-Tehran	589,242	518	5398	60
		Iraq-Baghdad	636,729	578	2904	32
20	Mencherz	Iran-Qom	7030	1.2	1042	38
		Iran-Tehran	10,886	2.4	1590	52
		Iraq-Baghdad	7397	2.4	734	65
21	Neshan	Iran-Qom	344,116	267	3256	69
		Iran-Tehran	394,394	307	4006	60
		Iraq-Baghdad				
22	Google Maps	Iran-Qom	115,675	84.2	3047	52
		Iran-Tehran	232,861	196	4480	78
		Iraq-Baghdad	211,289	183	2051	110
23	Radio Javan	Iran-Qom	326,995	199	3745	52
		Iran-Tehran	554,015	470	5221	78
		Iraq-Baghdad				
24	Spotify	Iran-Qom	345,047	201	5373	66
		Iran-Tehran	273,043	243	4156	81
		Iraq-Baghdad	175,090	155	1308	66
25	Canva	Iran-Qom	341,896	242	4917	50
		Iran-Tehran	553,189	466	4347	48
		Iraq-Baghdad	193,644	150	1893	57
26	Google Drive	Iran-Qom	142,811	117	3156	50
		Iran-Tehran	733,120	690	1937	63
		Iraq-Baghdad	169,532	145	1790	122
27	OneDrive	Iran-Qom	143,248	113	2957	83
		Iran-Tehran	120,416	109	3087	58
		Iraq-Baghdad	132,821	105	1282	63
28	Alibaba.com	Iran-Qom	272,121	195	6005	36
		Iran-Tehran	159,568	103	4361	19
		Iraq-Baghdad	166,402	132	2691	45
29	Digikala	Iran-Qom	380,549	327	3880	85
		Iran-Tehran	394,503	318	5578	49
		Iraq-Baghdad				
30	Torob	Iran-Qom	106,868	74.3	3417	49
		Iran-Tehran	228,344	173	4798	110
		Iraq-Baghdad				
31	LinkedIn	Iran-Qom	1081,869	970	2292	114
		Iran-Tehran	753,588	622	2790	114
		Iraq-Baghdad	499,624	508	1452	79
32	Instagram	Iran-Qom	365,061	270	4192	37
		Iran-Tehran	436,257	482	1495	41
		Iraq-Baghdad	1054,541	1110	5362	141
33	Facebook lite	Iran-Qom	298,403	216	7291	50
		Iran-Tehran	152,057	133	3498	40
		Iraq-Baghdad	102,368	88.4	1329	29
34	Facebook	Iran-Qom	301,761	230	5455	54
		Iran-Tehran	225,039	233	1488	29
		Iraq-Baghdad	439,253	447	3347	116
35	Google Play Store	Iran-Qom	105,015	90	2846	40
		Iran-Tehran	1580,145	1660	4410	90
		Iraq-Baghdad	450,922	479	1171	117
36	Bazaar	Iran-Qom	254,924	176	3559	52
		Iran-Tehran	1627,800	1300	2993	63
		Iraq-Baghdad				

**Table A2:** Dataset specification per capture.

#	File(Capture) Name	Apps in Capture	No. Packets	No. Bytes (MB)	No. Bi-Flows	Capture Duration (Minute)	Data repository or Identifier (DOI or Accession Number)
1	Qom-01	Copilot, Google Chrome, Google Drive	127,942	82.3	5655	762	Mendeley Data:Qom
2	Qom-02	MS Edge, OneDrive, Perplexity	129,705	85.3	2584	773	Mendeley Data:Qom
3	Qom-03	Duolingo, Eitaa, Opera Mini	77,684	41.7	6802	726	Mendeley Data:Qom
4	Qom-04	Elsa Speak, Gmail, Skype	95,835	71.8	1165	732	Mendeley Data:Qom
5	Qom-05	Email, Filimo, Mencherz	296,043	257	1246	756	Mendeley Data:Qom
6	Qom-06	Google Maps, Rubika, Snapp	287,009	221	3043	732	Mendeley Data:Qom
7	Qom-07	Canva, Neshan, Opera Mini	226,524	158	7393	770	Mendeley Data:Qom
8	Qom-08	Bazaar, Digikala, LinkedIn	176,615	151	1528	739	Mendeley Data:Qom
9	Qom-09	Alibaba.com, Copilot, Eitaa	102,853	71	1798	720	Mendeley Data:Qom
10	Qom-10	Google Chrome, Perpelexity, Torob	120,929	72.6	2513	730	Mendeley Data:Qom
11	Qom-11	Elsa Speak, Filimo, MS Edge	437,975	367	4542	849	Mendeley Data:Qom
12	Qom-12	Eitaa, Skype, Telegram	58,741	21.6	3956	756	Mendeley Data:Qom
13	Qom-13	Clash of Clans, Telegram, Youtube	310,469	159	12,998	1088	Mendeley Data:Qom
14	Qom-14	Elsa Speak, Filimo, MS Edge	307,907	259	2785	753	Mendeley Data:Qom
15	Qom-15	Gmail, Google Drive, Mencherz	120,923	97.9	1000	690	Mendeley Data:Qom
16	Qom-16	Email, OneDrive, Skype	37,868	22.3	909	722	Mendeley Data:Qom
17	Qom-17	Canva, Google Maps, Neshan	103,643	73.1	2414	751	Mendeley Data:Qom
18	Qom-18	Alibaba.com, LinkedIn, Rubika	703,966	544	3413	715	Mendeley Data:Qom
19	Qom-19	Digikala, Duolingo, Snapp	143,823	117	1860	744	Mendeley Data:Qom
20	Qom-20	Google Chrome, LinkedIn, Torob	90,455	61.2	2701	724	Mendeley Data:Qom
21	Qom-21	Copilot, Filimo, Skype	375,443	307	2013	719	Mendeley Data:Qom
22	Qom-22	Radio Javan, Spotify, Youtube	215,204	104	4433	725	Mendeley Data:Qom
23	Qom-23	Facebook, Google Play Store, Instagram	134,963	82.8	2848	737	Mendeley Data:Qom
24	Qom-24	Google Play Store, Radio Javan, Telegram	103,069	55.6	2582	963	Mendeley Data:Qom
25	Qom-25	Google Drive, Opera Mini, Perplexity	147,758	95	4951	747	Mendeley Data:Qom
26	Qom-26	Duolingo, Gmail, OneDrive	84,474	63.4	1402	764	Mendeley Data:Qom
27	Qom-27	Clash of Clans, Email, Snapp	162,698	73.9	3005	851	Mendeley Data:Qom
28	Qom-28	Google Maps, Radio Javan, Youtube	135,063	74.3	4034	795	Mendeley Data:Qom
29	Qom-29	Clash of Clans, Neshan, Spotify	356,433	162	3098	872	Mendeley Data:Qom
30	Qom-30	Facebook, Mencherz, Rubika	151,550	85.2	4151	859	Mendeley Data:Qom
31	Qom-31	Bazaar, Canva, Copilot	209,130	99.4	3692	855	Mendeley Data:Qom
32	Qom-32	Bazaar, Facebook, Spotify	212,286	118	3963	852	Mendeley Data:Qom
33	Qom-33	Facebook Lite, Google Play Store, Instagram	283,347	199	4891	734	Mendeley Data:Qom
34	Qom-34	Filimo, Instagram, Snapp	408,460	328	3355	783	Mendeley Data:Qom
35	Qom-35	Alibaba.com, Facebook Lite, Spotify	173,980	118	5734	742	Mendeley Data:Qom
36	Qom-36	LinkedIn, Radio Javan, Youtube	269,173	197	4751	696	Mendeley Data:Qom
37	Qom-37	Facebook Lite, Perplexity, Torob	196,633	124	4857	766	Mendeley Data:Qom
38	Qom-38	Bazaar, Canva, Rubika	373,649	277	4023	665	Mendeley Data:Qom
39	Qom-39	Clash of Clans, Digikala, Skype	386,603	288	3463	683	Mendeley Data:Qom
40	Qom-40	Facebook Lite, Telegram, Torob	135,526	87.6	5034	770	Mendeley Data:Qom
41	Qom-41	Google Chrome, Google Maps, OneDrive	129,945	94.6	2182	876	Mendeley Data:Qom
42	Qom-42	Elsa Speak, Neshan, Rubika	177,837	127	2080	832	Mendeley Data:Qom
43	Qom-43	Facebook, Google Drive, Youtube	266,295	188	5949	846	Mendeley Data:Qom
44	Qom-44	Duolingo, Skype, Spotify	153,523	107	4071	835	Mendeley Data:Qom
45	Qom-45	Email, Gmail, LinkedIn	323,064	289	1507	1074	Mendeley Data:Qom
46	Qom-46	CoinMarketCap, Duolingo, OneDrive	148,249	109	3180	936	Mendeley Data:Qom
47	Qom-47	CoinMarketCap, LinkedIn, Neshan	444,410	381	2376	1009	Mendeley Data:Qom
48	Qom-48	CoinMarketCap, Filimo, LinkedIn	419,286	359	1959	971	Mendeley Data:Qom
49	Qom-49	Digikala, Duolingo, LinkedIn	194,111	155	1682	1008	Mendeley Data:Qom
50	Qom-50	Digikala, Gmail, OneDrive	117,002	87.6	1318	969	Mendeley Data:Qom
51	Qom-51	Digikala, Email, Whatsapp Messenger	101,586	70	2432	959	Mendeley Data:Qom
52	Qom-52	CoinMarketCap, Gmail, Whatsapp Messenger	168,022	112	5906	1022	Mendeley Data:Qom
53	Qom-53	Email, Filimo, Whatsapp Messenger	237,693	182	1912	1002	Mendeley Data:Qom
54	Tehran-01	Facebook Lite, Google Chrome, Telegram	98,404	55.2	6156	615	Mendeley Data:Tehran #1
55	Tehran-02	Duolingo, Facebook Lite, MS Edge	146,270	113	2803	601	Mendeley Data:Tehran #1
56	Tehran-03	Gmail, Radio Javan, Skype	126,056	71.9	2504	564	Mendeley Data:Tehran #1
57	Tehran-04	Filimo, Google Play Store, OneDrive	765,734	643	1560	568	Mendeley Data:Tehran #1
58	Tehran-05	Elsa Speak, Instagram, Snapp	263,566	219	2243	554	Mendeley Data:Tehran #1
59	Tehran-06	Eitaa, Opera Mini, Perplexity	528,229	278	3119	565	Mendeley Data:Tehran #1
60	Tehran-07	Google Play Store, Torob, Youtube	269,888	264	2102	618	Mendeley Data:Tehran #1
61	Tehran-08	LinkedIn, OneDrive, Skype	284,802	168	1834	607	Mendeley Data:Tehran #1
62	Tehran-09	Digikala, Instagram, Telegram	249,291	197	1784	606	Mendeley Data:Tehran #1
63	Tehran-10	Clash of Clans, Google Chrome, Rubika	1133,301	733	12,604	639	Mendeley Data:Tehran #1
64	Tehran-11	Duolingo, Filimo, Perplexity	353,973	198	2319	527	Mendeley Data:Tehran #1
65	Tehran-12	Elsa Speak, MS Edge, Radio Javan	290,316	199	5349	603	Mendeley Data:Tehran #1
66	Tehran-13	Facebook Lite, Neshan, Youtube	222,296	148	1793	598	Mendeley Data:Tehran #1
67	Tehran-14	Bazaar, Google Play Store, Skype	449,502	320	2490	608	Mendeley Data:Tehran #1
68	Tehran-15	Copilot, Neshan, Torob	152,077	91.9	3172	662	Mendeley Data:Tehran #1
69	Tehran-16	Canva, Digikala, Google Maps	175,958	111	1777	574	Mendeley Data:Tehran #1
70	Tehran-17	Alibaba.com, Clash of Clans, Youtube	359,328	319	3899	565	Mendeley Data:Tehran #1
71	Tehran-18	CoinMarketCap, Filimo, Mencherz	117,507	67.6	2500	604	Mendeley Data:Tehran #1
72	Tehran-19	Digikala, Instagram, Telegram	121,463	104	1300	572	Mendeley Data:Tehran #1
73	Tehran-20	CoinMarketCap, Gmail, Google Play Store	449,149	435	2533	602	Mendeley Data:Tehran #1
74	Tehran-21	Google Chrome, Opera Mini, Telegram	163,032	92.3	5948	614	Mendeley Data:Tehran #1
75	Tehran-22	LinkedIn, OneDrive, Perplexity	179,865	111	2044	605	Mendeley Data:Tehran #1
76	Tehran-23	Google Maps, LinkedIn, Mencherz	29,904	21.1	929	574	Mendeley Data:Tehran #1
77	Tehran-24	Mencherz, MS Edge, Radio Javan	360,598	227	2443	584	Mendeley Data:Tehran #1
78	Tehran-25	Google Drive, Radio Javan, Rubika	308,099	182	4746	608	Mendeley Data:Tehran #1
79	Tehran-26	Clash of Clans, MS Edge, Telegram	78,909	44.6	1876	601	Mendeley Data:Tehran #1
80	Tehran-27	Copilot, Google Chrome, Google Maps	77,585	47.1	3377	601	Mendeley Data:Tehran #1
81	Tehran-28	Clash of Clans, Google Maps, Opera Mini	60,647	39.5	2181	589	Mendeley Data:Tehran #1
82	Tehran-29	Canva, Duolingo, Gmail	85,836	56.3	1726	589	Mendeley Data:Tehran #1
83	Tehran-30	Google Chrome, Instagram, Perplexity	243,690	219	5731	737	Mendeley Data:Tehran #1
84	Tehran-31	Alibaba.com, Clash of Clans, Youtube	87,238	68.9	2087	593	Mendeley Data:Tehran #1
85	Tehran-32	Eitaa, Gmail, Torob	257,920	231	948	616	Mendeley Data:Tehran #1
86	Tehran-33	Bazaar, Perplexity, Snapp	145,738	123	1266	612	Mendeley Data:Tehran #1
87	Tehran-34	Eitaa, Google Play Store, Rubika	270,156	247	1485	574	Mendeley Data:Tehran #1
88	Tehran-35	Filimo, Neshan, Radio Javan	203,896	189	1686	574	Mendeley Data:Tehran #1
89	Tehran-36	Clash of Clans, Digikala, Duolingo	132,994	82.3	3646	600	Mendeley Data:Tehran #1
90	Tehran-37	LinkedIn, MS Edge, Opera Mini	194,319	166	1913	644	Mendeley Data:Tehran #1
91	Tehran-38	Facebook Lite, Google Chrome, Telegram	165,434	134	2109	596	Mendeley Data:Tehran #1
92	Tehran-39	LinkedIn, MS Edge, Snapp	120,198	102	1048	609	Mendeley Data:Tehran #1
93	Tehran-40	Google Maps, Perplexity, Skype	57,541	29.9	1914	597	Mendeley Data:Tehran #1
94	Tehran-41	Canva, Copilot, Radio Javan	263,037	175	2115	599	Mendeley Data:Tehran #1
95	Tehran-42	Facebook, Google Maps, Snapp	222,881	180	2442	622	Mendeley Data:Tehran #1
96	Tehran-43	Google Play Store, Spotify, Torob	167,646	148	1504	640	Mendeley Data:Tehran #1
97	Tehran-44	Eitaa, Gmail, Neshan	132,110	86.2	1855	580	Mendeley Data:Tehran #1
98	Tehran-45	Filimo, Google Drive, OneDrive	235,289	192	1149	567	Mendeley Data:Tehran #1
99	Tehran-46	CoinMarketCap, LinkedIn, Whatsapp Messenger	61,018	31.5	1918	608	Mendeley Data:Tehran #1
100	Tehran-47	Email, Mencherz, Whatsapp Messenger	19,917	11.6	512	628	Mendeley Data:Tehran #1
101	Tehran-48	Google Chrome, Whatsapp Messenger, Youtube	328,281	294	2265	611	Mendeley Data:Tehran #1
102	Tehran-49	Canva, Copilot, Whatsapp Messenger	195,662	112	1150	573	Mendeley Data:Tehran #1
103	Tehran-50	Google Maps, Radio Javan, Whatsapp Messenger	84,278	57	1725	631	Mendeley Data:Tehran #1
104	Tehran-51	Facebook, Snapp, Whatsapp Messenger	65,751	42.4	1814	571	Mendeley Data:Tehran #2
105	Tehran-52	Google Play Store, Spotify, Whatsapp Messenger	98,755	70.4	846	570	Mendeley Data:Tehran #2
106	Tehran-53	Eitaa, Torob, Whatsapp Messenger	118,887	84.4	851	581	Mendeley Data:Tehran #2
107	Tehran-54	Elsa Speak, Neshan, Whatsapp Messenger	62,249	36.7	1036	603	Mendeley Data:Tehran #2
108	Tehran-55	Gmail, OneDrive, Whatsapp Messenger	101,370	61	1032	589	Mendeley Data:Tehran #2
109	Tehran-56	CoinMarketCap, Filimo, Google Drive	194,737	136	2700	622	Mendeley Data:Tehran #2
110	Tehran-57	Email, LinkedIn, Mencherz	34,540	23.8	885	772	Mendeley Data:Tehran #2
111	Tehran-58	Canva, Copilot, Radio Javan	176,213	125	2204	557	Mendeley Data:Tehran #2
112	Tehran-59	Facebook, Google Maps, Snapp	171,943	153	2218	561	Mendeley Data:Tehran #2
113	Tehran-60	Google Play Store, Spotify, Torob	181,431	170	995	572	Mendeley Data:Tehran #2
114	Tehran-61	CoinMarketCap, Digikala, Spotify	188,214	130	2557	580	Mendeley Data:Tehran #2
115	Tehran-62	Eitaa, Neshan, Torob	91,294	54.9	1430	629	Mendeley Data:Tehran #2
116	Tehran-63	Elsa Speak, Gmail, OneDrive	58,512	45.4	1495	638	Mendeley Data:Tehran #2
117	Tehran-64	Bazaar, Duolingo, Google Play Store	751,676	517	1298	651	Mendeley Data:Tehran #2
118	Tehran-65	Filimo, LinkedIn, Spotify	159,729	94.2	999	610	Mendeley Data:Tehran #2
119	Tehran-66	Eitaa, Elsa Speak, Torob	157,519	121	1736	665	Mendeley Data:Tehran #2
120	Tehran-67	Bazaar, Duolingo, Google Drive	321,288	253	2198	650	Mendeley Data:Tehran #2
121	Tehran-68	Filimo, LinkedIn, Spotify	274,875	196	1252	586	Mendeley Data:Tehran #2
122	Tehran-69	Eitaa, Elsa Speak, Torob	186,710	156	1601	742	Mendeley Data:Tehran #2
123	Tehran-70	Bazaar, Duolingo, Google Drive	489,586	373	1345	646	Mendeley Data:Tehran #2
124	Tehran-71	Filimo, LinkedIn, Spotify	362,324	245	1162	687	Mendeley Data:Tehran #2
125	Tehran-72	Eitaa, Elsa Speak, Torob	102,219	73.6	1380	598	Mendeley Data:Tehran #2
126	Tehran-73	Bazaar, Duolingo, Google Drive	430,762	322	1429	682	Mendeley Data:Tehran #2
127	Tehran-74	Eitaa, Filimo, LinkedIn	345,622	260	676	573	Mendeley Data:Tehran #2
128	Tehran-75	Duolingo, Spotify, Torob	38,328	24.6	1321	614	Mendeley Data:Tehran #2
129	Iraq-01	Facebook, Google Chrome, Instagram, Telegram, Youtube	340,307	345	1795	653	Mendeley Data:Iraq
130	Iraq-02	Facebook, Instagram, Telegram	154,825	154	1314	965	Mendeley Data:Iraq
131	Iraq-03	Instagram, LinkedIn	68,112	66.1	488	611	Mendeley Data:Iraq
132	Iraq-04	Facebook, Google Chrome, Google Maps, Google Play Store, Instagram, Telegram, Youtube	400,898	388	2885	935	Mendeley Data:Iraq
133	Iraq-05	Google Maps, Google Play Store, Instagram, Telegram, Youtube	488,547	502	1801	864	Mendeley Data:Iraq
134	Iraq-06	Gmail, Instagram	62,889	63.6	358	400	Mendeley Data:Iraq
135	Iraq-07	Gmail, Google Chrome, Telegram, Youtube	83,460	64.2	1663	818	Mendeley Data:Iraq
136	Iraq-08	Clash of Clans, Gmail, Instagram, Telegram, Youtube	1600,927	1500	4304	1290	Mendeley Data:Iraq
137	Iraq-09	Google Chrome, Google Drive, Instagram, Telegram, Youtube	312,704	302	2482	639	Mendeley Data:Iraq
138	Iraq-10	Facebook, Google Chrome, Google Drive, Google Play Store, Instagram, MS Edge, Telegram	222,614	160	3132	879	Mendeley Data:Iraq
139	Iraq-11	Clash of Clans, Google Maps, Instagram, Youtube	167,636	152	1223	706	Mendeley Data:Iraq
140	Iraq-12	Duolingo, Google Drive, Google Play Store	21,216	16.1	311	680	Mendeley Data:Iraq
141	Iraq-13	Gmail, Google Maps, Perplexity	63,890	54.9	468	842	Mendeley Data:Iraq
142	Iraq-14	Instagram, LinkedIn	124,923	120	620	665	Mendeley Data:Iraq
143	Iraq-15	Facebook Lite, Google Chrome, OneDrive	77,305	58.2	1252	759	Mendeley Data:Iraq
144	Iraq-16	Copilot, Google Maps, OneDrive	57,581	37.4	1014	683	Mendeley Data:Iraq
145	Iraq-17	Duolingo, Gmail, Google Drive	12,051	6.24	350	596	Mendeley Data:Iraq
146	Iraq-18	Facebook Lite, Google Play Store, Mencherz	26,234	17.2	520	521	Mendeley Data:Iraq
147	Iraq-19	Canva, Mencherz, Perplexity	30,442	16.2	664	517	Mendeley Data:Iraq
148	Iraq-20	Canva, Copilot, Mencherz	39,018	25.5	433	249	Mendeley Data:Iraq
149	Iraq-21	Canva, Mencherz, MS Edge	78,853	58.7	950	554	Mendeley Data:Iraq
150	Iraq-22	Facebook Lite, Mencherz, OneDrive	54,334	43.7	653	481	Mendeley Data:Iraq
151	Iraq-23	Canva, Gmail, Skype	161,101	67.3	1020	842	Mendeley Data:Iraq
152	Iraq-24	OneDrive, Perplexity, Youtube	68,610	55.7	583	383	Mendeley Data:Iraq
153	Iraq-25	Canva, Google Maps, Skype	71,022	34.8	634	508	Mendeley Data:Iraq
154	Iraq-26	Facebook, Google Maps, Perplexity	151,792	137	1205	1060	Mendeley Data:Iraq
155	Iraq-27	Copllot, Gmail. Google Play Store	45,335	30.2	935	592	Mendeley Data:Iraq
156	Iraq-28	Copilot,Gmail, Google play Store	26,859	16.9	445	379	Mendeley Data:Iraq
157	Iraq-29	Facebook, Google Maps, Perplexity	78,224	68.9	514	473	Mendeley Data:Iraq
158	Iraq-30	Facebook, LinkedIn, Mencherz	125,102	118	633	620	Mendeley Data:Iraq
159	Iraq-31	Canva, Google Drive, OneDrive	45,783	32.8	481	398	Mendeley Data:Iraq
160	Iraq-32	Facebook, LinkedIn, Mencherz	165,041	163	839	718	Mendeley Data:Iraq
161	Iraq-33	Canva, Google Drive, OneDrive	58,899	44.3	642	609	Mendeley Data:Iraq
162	Iraq-34	Facebook, Google Maps, Perplexity	87,130	70.7	1786	727	Mendeley Data:Iraq
163	Iraq-35	Google Drive, Google play Store, OneDrive	54,171	41.1	442	781	Mendeley Data:Iraq
164	Iraq-36	Duolingo, Google Drive, LinkedIn	148,149	137	874	960	Mendeley Data:Iraq
165	Iraq-37	Duolingo, Gmail, Google Play Store	144,857	123	1050	831	Mendeley Data:Iraq
166	Iraq-38	Gmail, Google Play Store, Spotify	105,835	96.5	576	849	Mendeley Data:Iraq
167	Iraq-39	Duolingo, Google Drive, Spotify	34,063	24.5	541	597	Mendeley Data:Iraq
168	Iraq-40	Google Drive, Perplexity, Spotify	102,560	88.4	762	615	Mendeley Data:Iraq
169	Iraq-41	Google Drive, LinkedIn, Mencherz	77,162	68.9	489	586	Mendeley Data:Iraq
170	Iraq-42	Duolingo, Google Drive, LinkedIn	186,623	184	908	695	Mendeley Data:Iraq
171	Iraq-43	Alibaba.com, Skype, Spotify	160,912	127	1919	620	Mendeley Data:Iraq
172	Iraq-44	Alibaba.com, Spotify, Whatsapp Messenger	69,980	45	985	769	Mendeley Data:Iraq
173	Iraq-45	Alibaba.com, Skype, Spotify	128,555	97.4	2117	602	Mendeley Data:Iraq
174	Iraq-46	Alibaba.com, Duolingo, Whatsapp Messenger	78,241	48.2	747	704	Mendeley Data:Iraq
175	ITC-Net-MingledApp_DetailedDescription	-					Mendeley Data: Supplementary

## Data Availability

Mendeley DataTehran Dataset #1 (Original data). Mendeley DataTehran Dataset #2 (Original data). Mendeley DataSupplementary Materials (Original data). Mendeley DataIraq Dataset (Original data). Mendeley DataQom Dataset (Original data).
